# Long-Range Activation of Systemic Immunity through Peptidoglycan Diffusion in *Drosophila*

**DOI:** 10.1371/journal.ppat.1000694

**Published:** 2009-12-18

**Authors:** Mathilde Gendrin, David P. Welchman, Mickael Poidevin, Mireille Hervé, Bruno Lemaitre

**Affiliations:** 1 Global Health Institute, EPFL, Lausanne, Switzerland; 2 Centre de Génétique Moléculaire (CGM), CNRS, Gif-sur-Yvette, France; 3 Institut de Biochimie et Biophysique Moléculaire Cellulaire, UMR 8619 CNRS, Université Paris-sud, Orsay, France; Stanford University, United States of America

## Abstract

The systemic immune response of *Drosophila* is known to be induced both by septic injury and by oral infection with certain bacteria, and is characterized by the secretion of antimicrobial peptides (AMPs) into the haemolymph. To investigate other possible routes of bacterial infection, we deposited *Erwinia carotovora (Ecc15*) on various sites of the cuticle and monitored the immune response via expression of the AMP gene *Diptericin*. A strong response was observed to deposition on the genital plate of males (up to 20% of a septic injury response), but not females. We show that the principal response to genital infection is systemic, but that some AMPs, particularly *Defensin*, are induced locally in the genital tract. At late time points we detected bacteria in the haemolymph of immune deficient *Relish^E20^* flies, indicating that the genital plate can be a route of entry for pathogens, and that the immune response protects flies against the progression of genital infection. The protective role of the immune response is further illustrated by our observation that *Relish^E20^* flies exhibit significant lethality in response to genital *Ecc15* infections. We next show that a systemic immune response can be induced by deposition of the bacterial elicitor peptidoglycan (PGN), or its terminal monomer tracheal cytotoxin (TCT), on the genital plate. This immune response is downregulated by PGRP-LB and Pirk, known regulators of the Imd pathway, and can be suppressed by the overexpression of PGRP-LB in the haemolymph compartment. Finally, we provide strong evidence that TCT can activate a systemic response by crossing epithelia, by showing that radiolabelled TCT deposited on the genital plate can subsequently be detected in the haemolymph. Genital infection is thus an intriguing new model for studying the systemic immune response to local epithelial infections and a potential route of entry for naturally occurring pathogens of *Drosophila*.

## Author Summary

Innate immunity is the first line of antimicrobial defence for vertebrates and the only immune response present in invertebrates such as the fruitfly *Drosophila*, which provides a powerful model system to study innate immunity. Interestingly, local infections of epithelia like the gut and, in our study, the genital tract, result not only in a local immune response, but in an immune response of the whole body. The latter seems to protect *Drosophila* against the potential spread of local infections. We have investigated the immune response to bacteria placed on the genitalia, at the entrance to both the genital tract and hindgut. This could be a natural entry route of pathogens, possibly linked to sexually transmitted infections. We observe a strong immune response to Gram-negative bacteria, mediated by the immune responsive Imd signalling pathway. This response depends on peptidoglycan, a crucial component of the bacterial cell wall, as pure peptidoglycan placed on the genitalia is sufficient to trigger a whole body immune response. Finally, we present strong evidence that peptidoglycan fragments within the genital tract or hindgut can cross these epithelia, enter the body cavity and thus induce a system wide immune response to a local infection.

## Introduction

Antimicrobial peptides (AMPs) are key components of innate immunity. They are ubiquitous throughout the animal and plant kingdoms, reflecting the importance of these molecules in host defence. In *Drosophila* over 20 AMPs, divided into seven classes, have been described [1]. These Insect AMPs are thought to be active against microbial membranes and exhibit specificity of activity against Gram-negative bacteria (e.g. Diptericin), Gram-positive bacteria (e.g. Defensin) or fungi (e.g. Drosomycin).

*Drosophila* AMPs are induced in the fat body, an analogue of the mammalian liver, in response to both septic injury and oral infection with certain bacteria. This response is referred to as the systemic response because it leads to secretion of AMPs into the haemolymph, which bathes all tissues. The systemic response has been well characterised and is regulated at the transcriptional level by the Toll and Imd pathways [2],[3]. The Toll pathway is induced by both Gram-positive bacteria and fungi, via circulating pattern recognition receptors, and leads to the activation of NF-κB proteins (Dif and Dorsal), controlling the production of AMPs active against fungi. In contrast, the Imd pathway mainly responds to Gram-negative bacterial infection and controls antibacterial peptide genes via the activation of the NF-κB-like protein Relish. PGRP-LC, one of several Peptidoglycan Recognition Proteins in *Drosophila*, acts as a membrane-bound pattern recognition receptor that activates the Imd pathway upon sensing of DAP-type Peptidoglycan (PGN) [4],[5],[6]. PGN is a cross-linked polymer which is an essential component of all bacterial cell walls. DAP-type PGN, which contains a diaminopimelic acid (DAP) moiety, is limited to Gram-negative bacteria and some Gram-positive bacilli, while the Lys-type PGN of most Gram-positive bacteria contains Lysine instead of DAP.

*Drosophila* AMP genes are also expressed in barrier epithelia such as the epidermis, reproductive system, respiratory tract and digestive tract, which are exposed to environmental microorganisms and/or indigenous flora [7],[8],[9]. This AMP synthesis is referred to as the local immune response, as opposed to the systemic response. It is important, in epithelia, to distinguish between constitutive and inducible AMP gene expression. Some AMP genes are expressed constitutively, in specific tissues, in the absence of infection. This is the case for *Drosomycin* in salivary glands and the female spermatheca [7], for *Drosocin* in the ovaries [10] and for *Cecropin* in the male ejaculatory duct [11]. This constitutive expression is not regulated by NF-κB pathways but by various tissue-specific transcription factors such as the homeobox-containing protein Caudal [12]. By contrast, the inducible local AMP gene expression is triggered upon infection by Gram-negative bacteria and is mediated by the Imd pathway [8],[13],[14]. For example, *Drosomycin* and *Diptericin* are induced in both trachea and gut in response to local infections by bacteria such as *Erwinia carotovora*. Like the systemic response, the local immune response is based on the recognition of Gram-negative PGN by PGRP-LC [15].

In addition to the immune response to infection, AMP expression has also been shown to be stimulated by mating, in females [16]. Surprisingly, this induction appears to be independent of any microbial elicitor. Indeed, experimental data support the idea that accessory gland proteins, including Sex Peptide, present in the sperm of males, are sufficient to induce a local AMP gene expression in the female genitalia. This immune response may limit the propagation of potential infectious agents just after copulation. This may be particularly important following female genital wounding, which has been shown to occur during copulation in *D. melanogaster* [17].

These recent observations have shown that the genes encoding AMPs are expressed in more sophisticated and differentiated patterns than were previously anticipated [9]. The expression of AMP genes appears to be specifically regulated both locally and systemically. In addition to some constitutive expression, they can be induced upon infection and upon mating. The starting point of this study was to investigate additional possible routes of infection and analyse whether Insects could sense the presence of bacteria through their cuticle, leading to expression of AMP genes. Here, we show that deposition of bacteria on the genital plate of males is sufficient to activate both systemic and local immune responses. Our results strongly suggest that the translocation of small fragments of PGN into the haemolymph triggers a protective systemic response upon genital infection.

## Results

### External genitalia application of bacteria activates an Imd pathway dependent immune response

**Figure 1:**
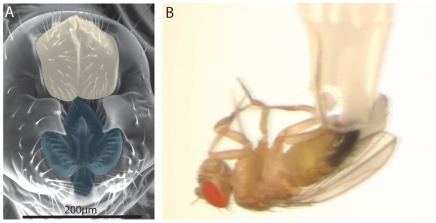
Infection of *Drosophila* males through the genitalia. **(A)** A scanning electron microscopy image of the male genital plate (kindly provided by Jagadeeshan and Singh). Note the complex cuticular structures surrounding the anal (yellow) and genital (blue) openings. Scale bar = 200µm. See also Jagadeeshan and Singh [46]. **(B)** GI of males was performed by depositing about 30nL of bacteria or microbial products on the genital plate using a 200µL tip as illustrated.

**Figure 2:**
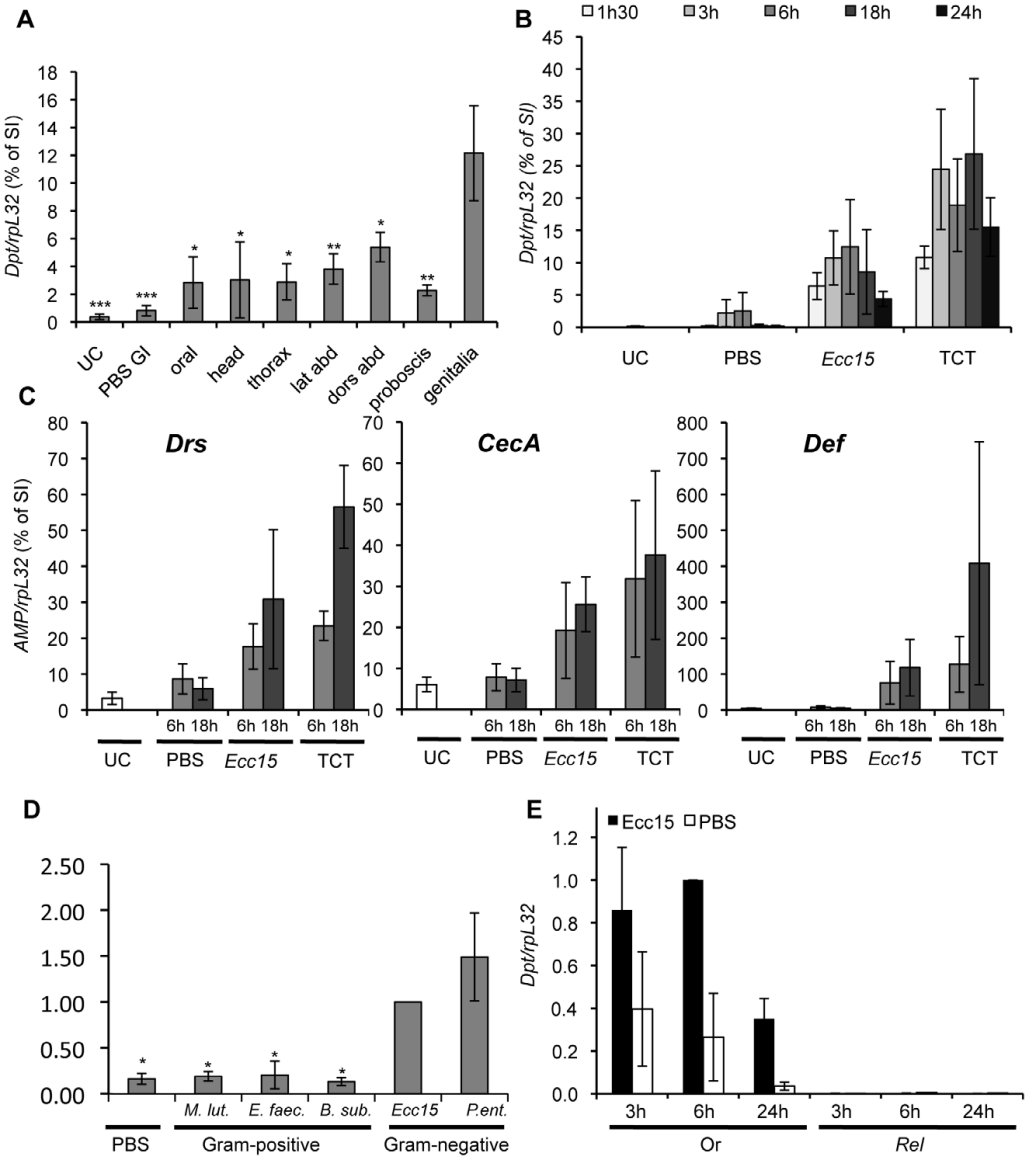
RT-qPCR experiments reveal that genital infection induces an immune response via the Imd pathway. **(A)** Expression profile of *Dpt* following deposition of *E. carotovora* bacteria (*Ecc15*) on various regions of the cuticle. *Dpt* was measured by RT-qPCR, 6h after deposition of around 30nL of bacteria (OD_600_ = 200) on various parts of the cuticle. Deposition of PBS on the genitalia (PBS GI) was used as a control. lat – lateral; dors – dorsal; abd – abdomen. **(B)** Time-course analysis of *Dpt* reponse after genital deposition of PBS, *Ecc15* or TCT. **(C)** Expression profile of *Drosomycin (Drs)*, *CecropinA (CecA)* and *Defensin (Def)* in flies collected 6h (light grey) or 18h (dark grey) after genital deposition of PBS, *Ecc15* or TCT. **(D)** *Dpt* expression in flies 6h after GI by various bacteria: *M. luteus* (*M. lut*), *E. faecalis* (*E. faec*), *B. subtilis* (*B. sub*), *E. carotovora* (*Ecc15*) and *P. entomophila* (*P. ent*). **(E)** Time-course analysis of *Dpt* expression in control OregonR (Or) and *Relish^E20^ (Rel)* flies after GI with *Ecc15*. Wild-type (Oregon^R^) flies were used, except where otherwise noted. In **A–C**, results are shown as a percentage of the *Dpt/rpL32* ratio of flies collected 6h after septic injury (SI) with *Ecc15* in the same experiment. In **D–E** the *Dpt/rpL32* ratio is normalised to the expression level 6h after *Ecc15* GI infection of wild-type flies. Data are the mean of 3 (**B, C, E**), 4 (**A, D**) or 8 (**A**: UC, PBS, dors abd, lat abd, genitalia) repeats, error bars show the standard error. In **A** and **D**, statistical analysis was performed using a Kruskal-Wallis test across all treatments (p = 0.016 in **A**, p = 0.009 in **D**), followed by a Tukey test for pair-wise comparisons of genital deposition of *Ecc15* to the other treatments (* p<0.05. ** p<0.01, ***p<0.001). UC – unchallenged, *Dpt* – *Diptericin*.

We wondered if flies are able to sense the presence of potentially infectious microorganisms in contact with their external cuticle. Entomopathogenic fungi have been shown to be able to cross the cuticle barrier and thereby activate an immune response [18], but to date bacteria have only been shown to activate an immune response by direct introduction into the haemolymph (septic injury) or by oral infection. To address this question, we deposited Gram-negative bacteria on various sites of the cuticle of adult males and monitored the systemic immune response 6h later, by the expression of the AMP gene *Diptericin* in whole flies. Deposition of a droplet of the Gram-negative bacteria *Erwinia carotovora carotovora 15* (*Ecc15*, OD_600_ = 200) on the head, thorax or abdomen, with a paintbrush, results in modest induction of *Diptericin* corresponding to less than 6% of the expression level observed after septic injury. In contrast, we observed that deposition of bacteria on the genital plate (see Figure 1A) reproducibly results in a substantial level of *Diptericin* expression, corresponding to 10 to 20% of the level obtained after septic injury (Figure 2A,B).

For all further experiments, genital infection (GI) was performed by applying 20–50nL of bacterial pellet using a 200µl pipette tip (see Figure 1B) depositing a droplet without touching the genital plate itself. A kinetic analysis reveals that *Diptericin* expression is already detectable at 1.5h post GI, reaches its maximum between 6 and 18h and decreases thereafter (Figure 2B). RT-qPCR analysis also indicates that GI triggers the expression of all the families of AMPs known to be induced in the systemic immune response (Figure 2C and Figure S1). Of all the AMP genes, *Defensin* exhibited the highest level of expression upon GI, reaching levels comparable to those observed in response to a systemic infection. Importantly, *Diptericin* expression in females, following an equivalent deposition of bacteria on the abdominal terminalia, was very low, with a maximum of 5% of the level observed after septic injury, indicating that the observed immune reactivity is specific to the male genitalia (data not shown). Since mating can induce wounding of the genital plate (at least in females [17]), we compared the response of mated to unmated males and found that the response was similar or slightly higher in unmated males (Figure S2). All subsequent experiments were performed with males from stocks, which were likely mated.

The genital plate is distinct from the other cuticle sites on which bacteria were deposited in that it includes openings to both the gut and genital tract. It should be noted however that other deposition sites included spiracles, opening into the trachea, and the proboscis, opening into the foregut. Thus the presence of openings into epithelial organs is not in itself sufficient to allow a systemic response to bacterial deposition on the cuticle.

Systemic expression of AMP genes is regulated by both the Toll and Imd pathways, which are activated by different classes of microbes in *Drosophila*. We investigated the range of bacteria to which a response to GI occurs and observed that AMP genes are induced only upon GI by Gram-negative but not by Gram-positive bacteria (Figure 2D and S1). This suggests that the Imd pathway mediates the response to GI and, indeed, *Diptericin* gene expression upon GI was abolished in *Relish^E20^* mutant flies that lack a functional Imd pathway (Figure 2E).

Our results indicate that male flies react strongly to the presence of bacteria deposited on the genital plate. The genital plate is a complex cuticular structure that provides many sites where bacteria could accumulate (see Figure 1A). Furthermore, since it includes both the anal and genital openings, it could provide a route of entry for potential pathogens into the digestive and/or genital tracts.

### The immune response to genital infection protects against colonisation of the haemocoel

To test if the genital plate is indeed a site of entry for bacteria into organs and into the haemolymph, we investigated the fate of living bacteria during GI. We monitored the fate of GFP expressing *Ecc15 (Ecc15-GFP)* deposited on the genital plate and observed that, after 9h, GFP was still present at the infection site in 90% of the flies. In contrast, only 50% of the flies still show GFP signal when bacteria were deposited on the side of the abdomen (data not shown). This suggests that the genitalia offer a better niche for the persistence of bacteria than the cuticle in general. We further observed that, after 24h, some flies which had had bacteria deposited on their abdomen exhibited bacteria on the genitalia, suggesting that bacteria washed from the body are able to infect the genitalia. Dissection of the genital tract of genitally infected flies revealed small quantities of fluorescent bacteria within the ejaculatory bulb and duct although the majority appears to remain on the external genitalia (data not shown).

**Figure 3:**
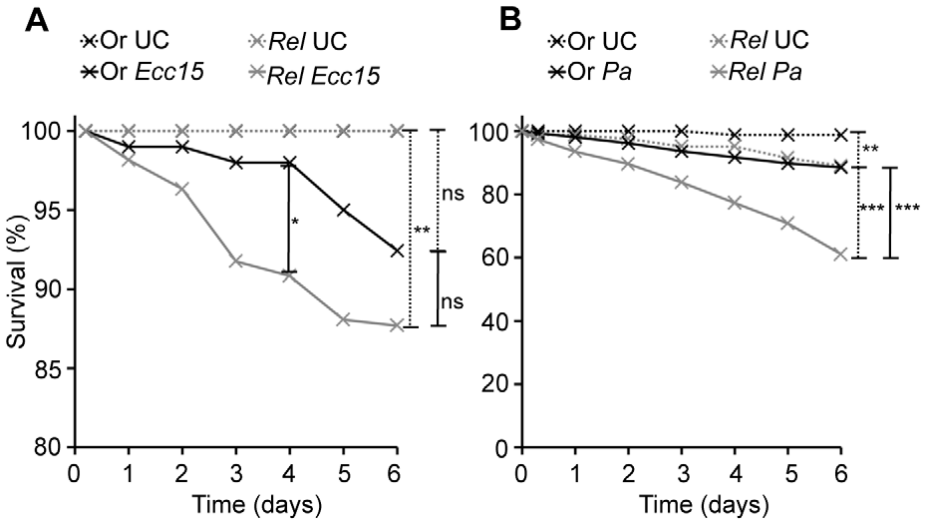
Immune deficient flies are more susceptible to genital infection. Survival analysis of wild-type (Or) and *Relish^E20^* (*Rel*) flies upon GI with *Ecc15* **(A)** and *P. aeruginosa* (*Pa*) **(B)**. Unchallenged (Or UC and *Rel* UC) flies were used as controls. The Kaplan-Meier log rank test was used to determine statistical significance. Dashed brackets represent the significance between unchallenged and infected flies and solid brackets between infected flies of different genotypes (* = p<0.05, ** = p<0.01, *** = p<0.001, ns = not significant). In **A**, *Rel Ecc15* is significantly different from Or *Ecc15* only after 4 days, but not later. The scale of the Y-axis in **A** has been modified to enable a better discrimination between the curves. Data are means of four independent experiments.

We did not detect any difference between *Ecc15-GFP* persistence on the outside of the genitalia in *Relish^E20^* and wild-type flies. However, by dissecting flies 24h after GI, we observed fluorescent bacteria in the body cavity of 19% of *Relish^E20^* flies (5/27), while no bacteria were found inside wild-type flies (n = 20). In order to verify the presence of live *Ecc15-GFP* we extracted haemolymph from genitally infected flies and plated the extracts, under Rifampicin selection for *Ecc15*. Live *Ecc15-GFP* were present in the haemolymph of 20% of *Relish^E20^* flies (4/20) but none were present in the haemolymph of wild-type flies (n = 20). Although a systemic immune response is already detected at 1.5h after GI, no bacteria were detected in the haemolymph at this time point, in either wild-type or *Relish^E20^* flies, by direct observation of fluorescence. We cannot exclude however that small numbers of bacteria, below our limit of detection, were present. Consistent with the presence of bacteria in their body cavity, we observed a weak but significant mortality after GI with *Ecc15* in *Relish^E20^* flies (Figure 3A). We also observed mortality in both wild-type and, more strongly, *Relish^E20^* flies after GI with a strain of *Pseudomonas aeruginosa* (CFBP2466), further suggesting that the genital plate provides a favourable route of infection in *Drosophila* (Figure 3B). These experiments demonstrate a role of the Imd pathway in controlling the progression of genital infections, preventing bacteria from entering the haemolymph.

### The immune response to genital infection is largely systemic

**Figure 4:**
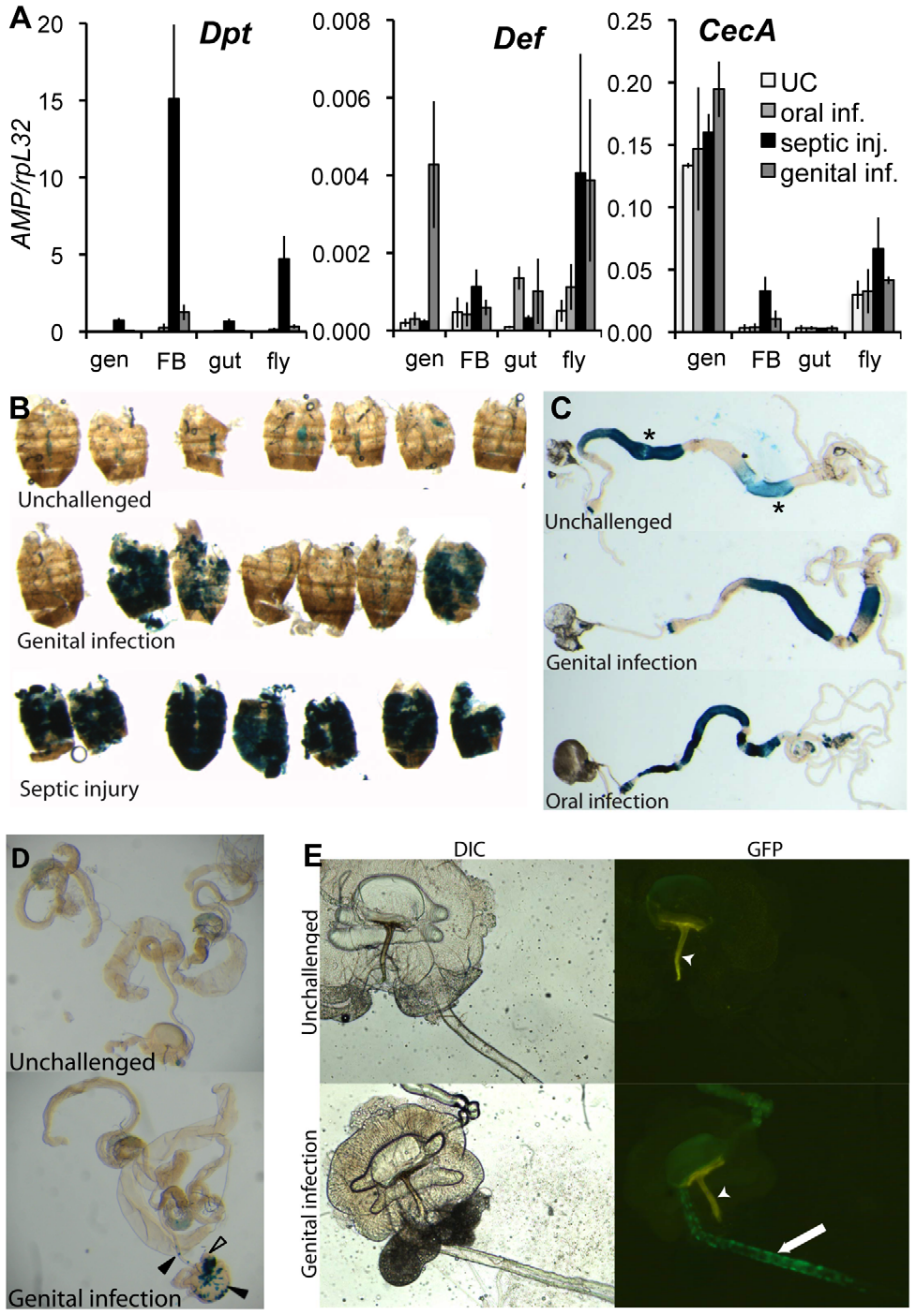
Genital infection induces both systemic and local immune responses. **(A)** RT-qPCR analysis of *Dpt*, *Def* and *CecA* expression in the genital tract (gen), the abdominal carcass (corresponding mostly to fat body, FB), the gut (including Malpighian tubules) and in whole flies, following oral infection, septic injury or GI with *Ecc15* (6h). **(B–D)** GI triggers *Dpt-lacZ* reporter gene expression in the fat body (**B**), the gut (**C**) and the genital tract (**D**). ß-galactosidase stainings were performed on fat body, gut and genital tract from wild-type flies (**B,C**) or *Gal^n1^* flies (deficient for the gene encoding endogenous *ß-galactosidase*) (**D**), carrying a *Dpt-lacZ* reporter gene. Flies were collected 6h after GI, septic injury or oral infection with *Ecc15*. GI triggers a strong expression of *Dpt-lacZ* in the fat body of around 20% of adults (**B**). A low level of ß-galactosidase activity (asterisks) corresponding to the endogenous *Gal* gene expression was observed in the gut of unchallenged flies (**C**). Upon GI *Dpt-lacZ* was induced weakly in the gut in around half of infected flies, while oral bacterial infection triggers a strong expression of the reporter in various gut segments (**C**). In the genital tract, GI triggers *Dpt-lacZ* in the ejaculatory bulb and internal ejaculatory duct (arrowheads, **D**). Note that the *Dpt-lacZ* reporter gene was also induced in an uncharacterised structure (empty arrowhead, **D**), attached to the ejaculatory bulb. This structure appears to be a convolution or overgrowth of the external ejaculatory duct leading from the ejaculatory bulb to the terminalia. Although it was not observed in all flies, its presence was not correlated with infection. **(E)** *Def-GFP* expression (green) is induced in the internal ejaculatory duct (arrow) 17h after GI with *Ecc15*. The apodeme (arrowhead) is strongly autofluorescent (yellow). In **A** the mean of three repeats is shown, error bars are standard error. In **B–E**, one representative experiment out of 3 repeats is shown.

Two bacterial strains, *Ecc15* in larvae and *Pseudomonas entomophila* in both larvae and adults, have been shown to be able to activate both local and systemic responses upon oral infection [13],[19]. To determine which tissues express AMPs upon GI with *Ecc15*, we used RT-qPCR to compare the expression levels of *Diptericin* in whole flies to those in dissected genital tracts, fat bodies (carcasses) and guts, 6h after infection. Figure 4A shows that *Diptericin* is most strongly expressed, upon GI, in the fat body. Using a *Diptericin-lacZ* reporter gene, we observed a strong fat body expression in about 20% of the males after GI (Figure 4B). Thus the *Diptericin* expression observed in whole flies is mostly due to a strong systemic induction in only 20% of the infected individuals. By contrast, *Diptericin* expression was induced in the fat body of 100% of flies subjected to septic injury.

In order to visualise the local immune response, we studied *Diptericin-lacZ* expression in the gut and genital tract. In contrast to oral infections, relatively little induction was observed in the gut (Figure 4C). In the genital tract, we monitored *Diptericin-lacZ* expression upon GI in *Gal^n1^* flies lacking the endogenous *ß-galactosidase* gene, as endogenous *ß-galactosidase* is expressed in the ejaculatory bulb. We observed a weak induction of the transgene in the seminal vesicle, the ejaculatory duct and ejaculatory bulb (Figure 4D). By qRT-PCR we observed that this induction is dependant on the Imd pathway (Figure S3).

We extended this analysis to other AMPs, using qRT-PCR and *AMP-GFP* reporter genes. Interestingly, we observed that GI specifically triggers a very strong induction of *Defensin* in the genital tract of males (Figure 4A) and that this induction is dependent upon the Imd pathway (Figure S3). Observation of flies expressing the *Defensin-GFP* reporter gene reveals that this expression is localised to the thin part of the ejaculatory duct (Figure 4E). We also confirmed previous observations that a *Cecropin A-GFP* reporter gene is strongly expressed in the ejaculatory duct in the absence of infection [8],[11] (data not shown), and expression analysis of the endogenous gene showed a slight induction upon GI (Figure 4A).

We conclude that deposition of bacteria on the genital plate is sufficient to activate both a systemic and a local immune response in *Drosophila* males. Most of the *Diptericin* expression observed in these flies corresponds to fat body expression. This indicates the existence of a link between genitalia and the fat body immune response.

### Genital application of purified Gram-negative PGN mimics a genital infection

The observation that deposition of bacteria on the genital plate induces a systemic immune response could be explained either by the invasion of haemolymph by bacteria or by the existence of an immune signal between genitalia and the fat body. Recently, it has been proposed that PGN translocation through the gut could be a mechanism that activates the systemic immune response upon oral bacterial infection with *P. entomophila* or *Ecc15* [15]. Our observation that systemic *Diptericin* expression was detectable before bacteria were observed in the haemolymph of even immune deficient flies, suggests that a similar mechanism explains the systemic response to GI.

**Figure 5:**
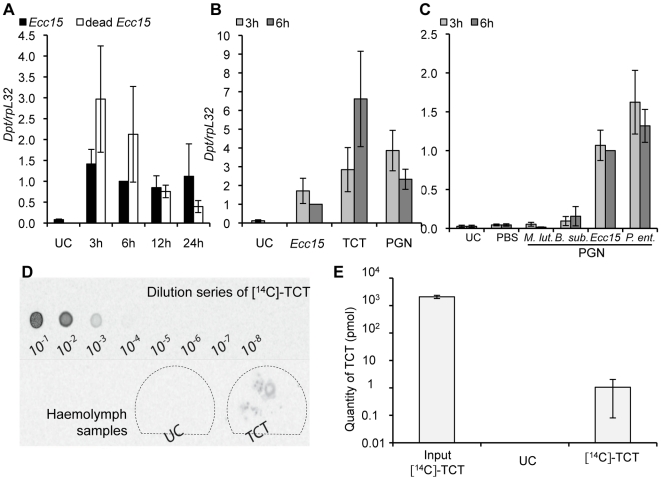
TCT is sufficient to induce an immune response and is translocated into the haemolymph. **(A–C)** RT-qPCR analysis of *Dpt* expression following deposition on the genital plate of live or dead *Ecc15* (**A**), PGN and TCT compared to *Ecc15* (**B**) or PGN extracted from various bacterial species compared to PBS (**C**). Data are means of three repeats, error bars are standard errors. UC: Unchallenged, *M.lut.: M. luteus*, *B.sub.: B. subtilis*, *Ecc15: E. carotovora*, *P.ent. : P. entomophila*. The *Dpt/rpL32* ratio is shown normalised to expression in flies 6h after genital challenge with either live *Ecc15* (**A,B**) or with *Ecc15* PGN (**C**). Wild-type (Oregon R) flies were used, except in **B**, where the flies used were the progeny of *da-Gal4* crossed to *w^1118^*. **(D)** [^14^C] labelled TCT was deposited on the genital plates of flies and haemolymph extracted two hours later. This experiment was repeated three times. Depicted is a representative scan of a phosphor-imager screen exposed to filter papers containing: *Upper panel:* 1µL drops of serial 1/10 dilutions of the [^14^C]-TCT solution deposited on the flies. There is signal saturation in the 10^−1^ drop. *Lower panel:* Haemolymph of unchallenged flies (UC) or flies where [^14^C]-TCT was deposited on the genital plate (TCT). The dashed lines represent the form of the filter papers. **(E)** Quantification of [^14^C] present in extracted haemolymph. Raw data were obtained as a percentage of the intensity of the 10^−2^ drop, and are presented here as the quantity of TCT (pmol) calculated from the known concentration of the [^14^C]-TCT source and the volume used to infect flies. Data shown are the mean of 3 repeats, error bars show standard error.

To distinguish between direct sensing of bacteria in the haemolymph and remote sensing of bacteria present in the genital tract and/or hindgut, we compared the systemic immune response, as monitored by the level of *Diptericin* expression, upon GI with living or dead bacteria. Figure 5A shows that dead bacteria activate a systemic immune response with a similar, or higher, efficiency to live bacteria. This demonstrates that the induction of a systemic response is not mediated by the crossing of the epithelial barriers by live bacteria but rather results from the release of bacterial elicitors. Monomeric or polymeric DAP-type PGNs of Gram-negative bacteria are the most potent inducers of the Imd pathway. This prompted us to analyze the effect of PGN deposition on the genital plate. As shown in Figure 5B, deposition of highly purified PGN from *Ecc15* activates a systemic immune response to the same, or greater, extent as live bacteria. Comparing the responses to PGN from different bacteria we found that DAP-type PGNs of Gram-negative *Ecc15* or *P. entomophila* induce a strong response, whilst PGNs from Gram-positive bacteria, either DAP-type from *B. subtilis* or Lys-type from *M. luteus*, activate little or no response (Figure 5C).

The GlcNAc-MurNAc(anhydro)-L-Ala-γ-D-Glu-*meso*-DAP-D-Ala monomer, also known as tracheal cytotoxin (TCT), was previously identified as the minimum PGN motif capable of efficiently inducing the Imd pathway [5],[6]. TCT provides an ideal “signature” of Gram-negative bacteria since this muropeptide is continuously released during cell growth and division as a result of PGN recycling [20]. We observed that GI with TCT strongly induces AMP genes (Figures 2B and C). It has been proposed that TCT released by cleavage of the PGN polymer can cross epithelia to activate an immune response. If this were the case, then TCT should induce a stronger systemic immune response than polymeric PGN. To compare the immune response to TCT or polymeric PGN, we deposited around 30nL of HPLC-purified TCT or PGN on the genital plate at (monomer equivalent) concentrations respectively of 1mmol.L^−1^ and 5mmol.L^−1^. Injecting serial dilutions of TCT or PGN, starting at these concentrations, the immune response to PGN is always the same or higher than that to TCT (Figure S4). By contrast, the response to genital deposition of 1mmol.L^−1^ TCT was particularly strong, three times the response to genital deposition of 5mmol.L^−1^ PGN at the 6h time point (Figure 5B). These results show that the systemic activation of AMP genes in response to GI can be mediated through the sensing of TCT or small fragments of PGN and does not require the presence of live bacteria. They further suggest that this sensing takes place in the haemolymph.

### Imd pathway regulators limit the immune response to genital infection

**Figure 6:**
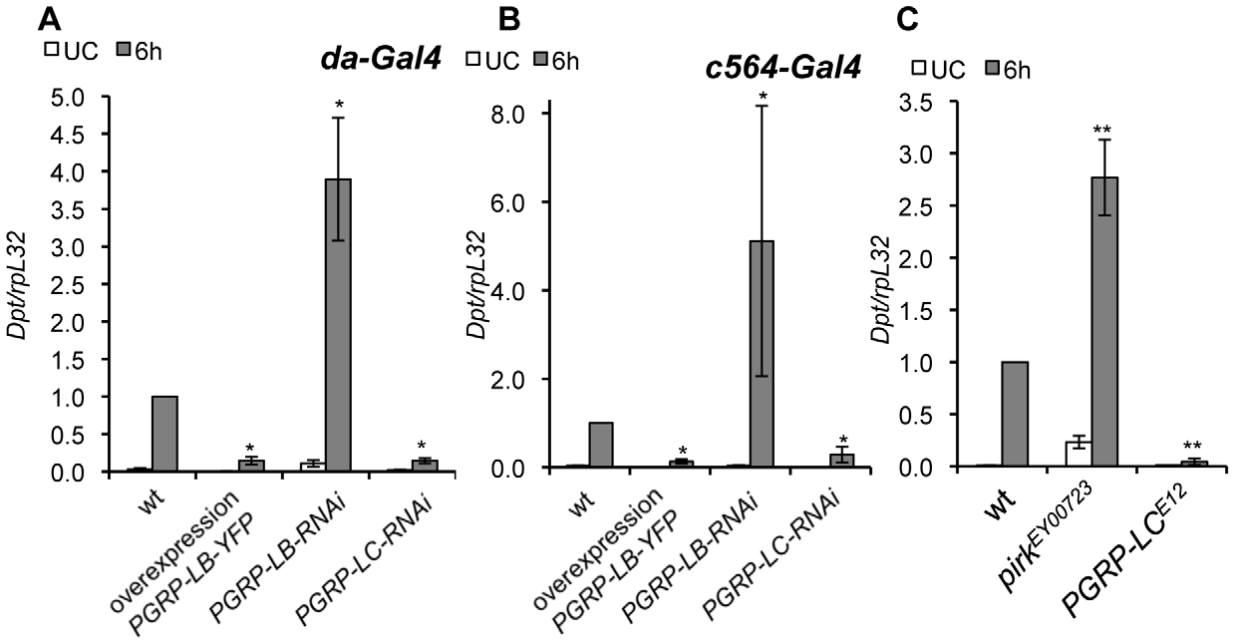
Pirk and PGRP-LB downregulate the immune response to genital infection. **(A,B)** Expression of *Dpt* in whole flies either unchallenged (UC) or 6h after genital deposition of TCT. Induction in control flies is compared to that in flies subjected to RNAi of *PGRP-LB* or *PGRP-LC* or overexpression of *PGRP-LB*, under the ubiquitous *da-Gal4* driver (**A**) or the tissue-specific fat body and haemocyte driver *c564-Gal4* (**B**). Similar results were obtained with two independent insertions of the *PGRP-LB* RNAi construct (R3 shown; R1 not shown). **(C)** Expression of *Dpt* in whole flies 6h after GI with *Ecc15*. Induction in control (Or) flies is compared to that in flies mutant for *pirk (pims^EY00723^)* or *PGRP-LC* (*PGRP-LC^E12^*). The *Dpt/rpL32* ratio is shown normalised to expression in GI wild-type flies (progeny of *da-Gal4* crossed to *w^1118^* in **A**, progeny of *c564-Gal4* crossed to *w^1118^* in **B**, Oregon in **C**). Statistical significance was determined by the Mann-Whitney U test where appropriate (* = p<0.05, ** = p<0.01). Data are the mean of 4 repeats and error bars are standard errors.

Recent studies in *Drosophila* have revealed that multiple levels of regulation limit Imd pathway activity and prevent excessive or prolonged immune activation [3]. A key role in bacterial tolerance of the gut has been attributed to the amidase PGRPs, PGRP-SC1 and PGRP-LB, as they are proposed to scavenge PGN released by gut microbes [15],[21]. An additional negative regulator, Pirk (also named Pims or Rudra), has been recently identified as an Imd immune responsive factor which removes the receptor PGRP-LC from the membrane, thereby shutting down Imd signalling [22],[23],[24]. We investigated the roles of PGRP-LB, Pirk and PGRP-LC in the immune response upon GI. As shown in Figure 6A, *Diptericin* was induced, upon deposition of TCT on the genitalia, at a higher level in flies subjected to ubiquitous *PGRP-LB* RNAi (with a *da*-*Gal4* driver) than in control flies. Furthermore, ubiquitous overexpression of *PGRP-LB* suppresses *Diptericin* induction by TCT. Specific overexpression of *PGRP-LB* in the fat body and haemocytes (with a *c564-Gal4* driver) also suppressed the induction of *Diptericin* by TCT (Figure 6B). This result demonstrates that the presence of the amidase PGRP-LB in the haemolymph compartment blocks the systemic immune response to GI, presumably by degrading fragments of PGN entering the haemolymph. Interestingly, RNAi of *PGRP-LB* in the fat body (driven by *c564-Gal4*) has a less reproducible effect than ubiquitous RNAi of *PGRP-LB* (Figure 6A,B), suggesting that PGRP-LB normally plays a more important role outside the haemolymph, in limiting the availability of PGN fragments capable of entering the haemolymph. However, the lack of a Gal4 driver expressed specifically in the male genital tract prevented us from testing whether it is in this tissue that PGRP-LB is required to limit the systemic response to GI. In keeping with the model of TCT entering the haemolymph, we observed that selective depletion of PGRP-LC in the fat body blocks the systemic immune response to TCT (Figure 6B). Similarly, *PGRP-LC^E12^* mutant flies show no systemic response to GI with *Ecc15* (Figure 6C). Finally, we observed a higher immune response to GI in *pirk* deficient flies, the level of *Diptericin* expression being 2.5 fold higher than in wild-type flies (Figure 6C).

We conclude that the activity of the Imd pathway is required in the haemolymph for a systemic response to genital infection and that this is blocked by the presence of a PGN degrading enzyme. As was shown for the gut immune response, the immune response to GI is tightly regulated, being limited by the Imd pathway modulators PGRP-LB and Pirk.

### Radiolabelled TCT deposited on the genitalia enters the haemolymph

To strengthen the model that translocation of TCT into the haemolymph is responsible for the systemic immune response to genital deposition of PGN or TCT, we analysed whether traces of TCT could be detected in the haemolymph following its deposition on the genital plate. To this end, we deposited [^14^C]-radiolabelled TCT on the genital plate of males and tested for the presence of radioactivity in the haemolymph. Around 30nL of TCT was deposited on the genitalia of 100 flies and haemolymph samples were carefully collected 2h later (shortly after the systemic immune response is first detected) and deposited on a filter paper. Figure 5D shows that a radioactive signal could be detected in the haemolymph and Figure 5E indicates that about 1/1000^th^ of the radioactivity deposited on the genitalia was recovered in the haemolymph 2h later. This is consistent with the observation that injection of 1µmol.L^−1^ TCT provokes a similar level of systemic immune response to genital deposition of 1mmol.L^−1^ TCT (Figure S4). RT-qPCR analysis revealed that *Diptericin* was expressed to similar levels in flies challenged with radioactive or non-radioactive TCT (data not shown). Since the [^14^C]-TCT used was radiolabelled at the level of the DAP, this experiment shows that [^14^C]-TCT or fragments of TCT containing the DAP are indeed translocated from the genital plate to the haemolymph.

## Discussion

In this paper, we show that the deposition of bacteria on genitalia is sufficient to trigger both local and systemic expression of AMP genes in *Drosophila* males. Of all the sites where bacteria were deposited, the immune response was the greatest in the case of the genitalia. This demonstrates an unexpected immune reactivity to the presence of bacteria on the genitalia, that probably relates to the possibility of pathogen entry via this route. Exploiting this new mode of infection, we also provide strong evidence that a systemic immune response can be activated at a distance by the presence of bacteria on the genitalia, through the diffusion of small fragments of PGN. Thus our study not only provides information on the mechanisms used to prevent microbial infection of the genital tract, but also describes a novel mode of infection, via the genitalia, which offers new opportunities for the study of the long-range activation of *Drosophila* immunity.

Although the integument constitutes a formidable barrier with the outside world, there are weak points in the surface that parasites and pathogens might be expected to target, such as the gut, trachea and reproductive organs [25]. Thus the genital plate, with openings to the gut and genital tract, seems a likely route of pathogen entry [26]. The convolutions of the cuticular structures of the genital plate may facilitate the persistence of bacteria at this site, reducing the efficiency of cleaning by leg brushing, a typical grooming behaviour of the fly. This is corroborated by our observation that bacteria deposited on this structure persist longer than those deposited elsewhere on the cuticle. We observed that deposition of Gram-negative bacteria on the genital plate is sufficient to induce AMP gene expression locally in both the genital tract and hindgut. Due to the proximity between anal and genital openings, it was not possible to distinguish whether GI results in infection of the genital tract, anus or both. However, since we observe that deposition of bacteria triggers stronger expression of AMP genes in the genital tract than in the gut, the genital tract could well be the main site of infection. Our study reveals for the first time that *Defensin*, an antibacterial peptide with activity against Gram-positive bacteria, is strongly induced in the genital tract upon GI with a Gram-negative bacterium, under control of the Imd pathway. Expression of the analogous vertebrate *Defensins* has also been reported in the reproductive tract and seminal fluid of male Rats, Mice and Humans, where it is proposed to play a role in the protection of germ cells [27].

Whereas we observed no significant immune response to bacterial deposition on the genitalia of female *Drosophila*, mating has been shown to stimulate the expression of AMP genes in females, as a result of accessory gland proteins present in the male seminal fluid [16]. These results support the theory that mating is a significant cause of infection for *Drosophila* in the wild [26]. In Insects generally, a wide range of infections have been shown to be transmitted during copulation [28] and potential pathogenic bacteria have been reported on male sexual organs [29]. In *Drosophila*, transfer of the entomopathogenic bacterium *Serratia marcescens* from contaminated males to females during courtship and mating has been observed in an experimental setting [30]. Our observations showing a high immune response to the presence of bacteria on the male genitalia suggest that the genital tract provides an entry route for bacteria in both sexes. It is intriguing that the two sexes seem to show immune responses to different genital stimuli. Immune differences between the sexes are commonly observed in Insects [31],[32] and it has been suggested that in some cases this is an efficiency measure since infections are more reliably coupled to mating in females than males [26]. This is taken to the extreme in the bedbug, *Cimex lectularius*, where the female has a specific immune organ developed in response to traumatic insemination [26]. Although traumatic insemination has not been reported in *Drosophila melanogaster*, the male is known to inflict genital wounds during mating [17], thus infection may be reliably coupled to mating, leading to the evolution of an immune response to copulation. That males show a direct response to bacteria on the genitalia, rather than to copulation, could be explained by a reduced risk of infection associated with mating in males and an advantage to maintaining clean genitalia, thereby avoiding transmission of bacteria to mated females.

To date, two methods, septic injury and oral ingestion of certain bacterial strains, have been shown to activate systemic expression of AMP genes in *Drosophila* in response to bacterial infection. In this study, we report a third mode of activation of the systemic immune response by showing that deposition of bacteria on the male genital plate results in an immune response in the fat body, without the presence of bacteria in the haemolymph. This shows that the genitalia allow the passage of ‘early-warning’ signals that recruit and activate haemolymph-based effector systems (see below). As has been observed for oral infections [8],[13], the immune response to GI is mediated by the Imd pathway and activated by DAP-type PGN of Gram-negative bacteria. The observation that DAP-type PGN of the Gram-positive *B. subtilis* is a less potent inducer might be explained by the absence of TCT and/or the high proportion of amidated DAP in *Bacillus* PGN. Interestingly, systemic *Diptericin* expression was observed in only 20% of GI treated males, all of these exhibiting a strong fat body expression. This suggests the existence of a threshold, with an all or nothing response. This clearly contrasts with the uniform immune response following septic injury. This threshold response is probably linked to the existence of multiple negative feedback controls such as PGRP-LB and Pirk, that regulate the Imd pathway and prevent its activation. We can further speculate that the threshold response depends upon the ability of bacteria to colonise the genital tract. An important barrier role of the immune response to genital infection is demonstrated by our observations that the haemocoel of *Relish^E20^*, but not wild-type, flies can be colonised by genitally deposited *Ecc15* and that *Relish^E20^* flies exhibit significantly higher lethality than wild-type flies upon GI with a strain of the opportunistic bacterium *P. aeruginosa*.

Although much progress has been made in our understanding of the regulation of innate immunity, it remains to be determined how microbe-derived molecules activate the immune system under physiological conditions. In other systems small muropeptide fragments of PGN, notably TCT, have been shown to act as diffusible signalling molecules. For example, TCT released by the symbiont *Vibrio fisheri* is involved in the differentiation of the light organ of the squid *Euprymna scolopes* [33]. Small muropeptides were also identified as products of the intestinal flora that induce the genesis of lymphoid follicles in the gut of Mice, through activation of the Nod1 pathway [34]. Although the intracellular receptors Nod1 and Nod2 are known to be activated by small PGN fragments, how these ligands reach the intracellular compartment is unclear. Some progress has been made recently, with the identification of a transmembrane receptor, Pept1, as a possible transporter of the Nod2 ligand, muramyl dipeptide [35]. Alternately, another recent study reports that the clathrin and dynamin dependent endocytosis pathway is a key component in the activation of the Nod2 pathway [36].

We have previously suggested a model whereby long-range activation of the systemic immune response in *Drosophila* is mediated by the translocation of small PGN fragments from the gut lumen to the haemolymph [15]. This view was supported by the observation that ingestion of monomeric PGN can stimulate a strong systemic immune response in *PGRP-LB* RNAi flies, which have reduced amidase activity and are unable to degrade PGN to its non-immunogenic form. Our present study of GI further supports this model of long-range activation of the immune system by PGN translocation. Deposition of PGN or TCT on the genitalia is sufficient to induce a systemic immune response and, in agreement with a model that involves transport of PGN, we observed that TCT activates a stronger systemic immune response than polymeric PGN. That the systemic immune response to GI requires PGRP-LC expression in the fat body, and is suppressed by amidase activity in the haemolymph, suggests that this response is mediated by PGN fragments present in the haemolymph. Finally, GI with radiolabelled TCT has allowed us to show for the first time that TCT (or a fragment thereof) can be found in the haemolymph shortly after genital deposition and that its presence correlates with the activation of an immune response. It seems likely that the detected molecules are intact TCT, since fragments of TCT are insufficient for immune activation via PGRP-LC [5]. These results demonstrate that TCT is indeed translocated into the haemolymph, although do not exclude that other signalling mechanisms contribute to the systemic immune response to local infections.

Although TCT has been shown to be a diffusible signalling molecule in other systems, few studies have addressed the extent to which the gut and genital epithelial barriers are permeable in *Drosophila*. Interestingly, some of the male-derived Accessory Gland Proteins, transferred to the female reproductive tract during mating, access presumptive target tissues bathed by the haemolymph [37]. Further, some have been shown to directly traverse the female reproductive tract and enter the haemolymph by crossing the ventral intima of the posterior vaginal wall [38]. In spite of this apparent permeability, PGN applied to the genitalia of females does not activate an immune response, underlining the difference between the genital tracts of male and female *Drosophila*. It remains possible that PGN fragments are translocated into the haemolymph by endocytosis or by specific transporters as has been suggested for ligands of the Nod2 pathway. Future work should decipher the mechanism, passive or active, of PGN translocation as well as the site of translocation. By contrast to oral infection, which depends upon the complex physiological regulation of feeding, GI is easy to control, since bacteria or PGN are applied directly to the genitalia. Thus GI provides a simple and reproducible mode of infection for further study of the long-range activation of the systemic immune response.

## Materials and Methods

### Fly stocks

Oregon^R^ flies were used as wild-type controls unless otherwise indicated. *Relish^E20^ (e^+^*, *Rel^E20^)*, *PGRP-LC^E12^* and *pirk* (*pims^EY00723^*) are described elsewhere [23],[39],[40]. *Gal^n1^* is a null mutation in the *ß-galactosidase* gene of *Drosophila* [41]. The *Gal4* lines used in this study specifically express GAL4 constitutively (*da-Gal4*) or in adult fat body and haemocytes (*c564-Gal4*) [42],[43]. *Defensin-GFP*, *Cecropin A-GFP* and *Diptericin-lacZ* strains were described previously [44].

The *UAS-PGRP-LB-IR* (insertion R1 and R3) and *UAS-PGRP-LC-IR1* inverted repeat RNAi stocks were generated by Ryu Ueda and are described elsewhere [15]. A full-length cDNA of *PGRP-LB* (using the *CG31217*_cDNA gold LD43740 from DGRC) was placed downstream of the UAS sequence using the pUASt vector. The *UAS-PGRP-LB-YFP* transgene was obtained by a fusion of YFP to the C-terminus of PGRP-LB inserted in the pDONR221 Gateway entry clone (Invitrogen) and finally subcloned in the pTWG transgenesis vector. RNAi and overexpression experiments were controlled by crossing the *Gal4* drivers to *w^1118^*, the strain in which the UAS construct insertions were generated. F1 progeny carrying both the *UAS* construct and the *Gal4* driver were transferred to 29°C at late pupal stage for optimal efficiency of the UAS/Gal4 system. *Drosophila* stocks and crosses were maintained at 25°C in yeasted tubes containing corn-meal fly medium [44].

### Bacterial stocks

All bacteria were stored as frozen stocks (15% DMSO) and cultured on LB-Agar plates and in LB medium. *Ecc15* and *Ecc15-GFP* strains are Rifampycin resistant and were described previously [13]. They were grown overnight at 29°C (without selection) and used as pellets of OD_600_ = 200 and 250 respectively [44]. The *Pseudomonas aeruginosa* strain 2466 (Collection Française de Bactéries Phytopathogènes, INRA, Angers, France) was used for survival experiments. It was grown at 37°C overnight and used as pellets of OD_600_ = 150–200. Additional bacteria, *Bacillus subtilis*, *Pseudomonas entomophila* [19] and *Micrococcus luteus*, were grown at 29°C and used as pellets of OD_600_ = 200. Pellets were not washed prior to use. Dead bacteria were produced by heating for 5 minutes at 95^o^C.

### Infection and survival experiments

Septic injuries were performed by pricking adults in the thorax with a thin needle dipped into a concentrated bacterial pellet [44]. For Figure 2A, bacterial depositions were performed using a paintbrush dipped into a bacterial pellet. For all other experiments, GI was performed by touching a 200µL pipette-tip containing 10µL of bacterial pellet or 5µL of PGN/TCT to the tip of the abdomen, thereby depositing a small droplet (20–50nL) covering the whole genital plate (see Figure 1B). Infected males were subsequently maintained at 29°C in tubes without yeast in the absence of females. In the case of the experiment using [^14^C]-TCT, each fly was maintained in a separate vial to prevent cross-contamination. For each bacterial strain, four independent survival experiments were performed with at least 20 flies per genotype, in replicates of 10–20 flies. Survival was scored every 24h.

### RT-qPCR

*AMP* and *rpL32* mRNA quantification by RT-qPCR was performed as described [44]. All expression data are given as a ratio of the expression level of the invariant mRNA *rpL32*. Each experiment was performed with approximately 20 flies for each genotype.

### Imaging

For GFP observation, flies were dissected in PBS and either directly observed under a Leica MZ16F dissecting microscope, or mounted in PBS for imaging with a Zeiss Axioimager Z1. β-galactosidase was visualised by X-gal staining, as previously described [44], followed by mounting in a 50∶50 mix of ethanol and glycerol. Images were captured with a Leica DFC300FX camera and Leica Application Suite or with an Axiocam MRn camera and Axiovision respectively.

### PGN and TCT

All PGN was prepared as described in Leulier et al, 2003 [4]. Each PGN was used at a monomer equivalent concentration of 5mmol.L^−1^. For production of TCT, *E. coli* PGN was purified from the BW25113 Δ*lpp*::Cm^R^ strain that does not express the Braun lipoprotein and digested by SltY lytic transglycosylase [5]. Radiolabelled *E. coli* PGN was obtained by incorporation of *meso*-[^14^C] DAP (11.6 kBq.nmol^−1^) into the FB8 *lysA::kan* strain grown in M63 minimal medium supplemented with 0.2% glucose and 100µg.ml^−1^ of lysine, threonine and methionine [45]. Radiolabelled [^14^C]-TCT, produced by digestion of the resulting material with SltY, was purified by HPLC and its specific activity was estimated as 2kBq.nmol^−1^. Both TCT and [^14^C]-TCT were used at a concentration of 1mmol.L^−1^.

### Haemolymph extraction

Haemolymph was extracted manually using a Nanodrop microinjector (Nanoject™) [44]. A glass needle containing approximately 100nL of protease inhibitors (1× Complete Mini, Roche, in PBS) was used to prick the flies in the dorsal thorax, inject 25nL of the inhibitor and immediately extract as much haemolymph as possible (through the same wound). For the experiment to detect [^14^C]-TCT, the dorsal thorax was washed, prior to haemolymph extraction, with a filter paper dipped in water to avoid contamination by [^14^C]-TCT potentially present on the cuticle. For each fly, the extracted haemolymph (and remaining protease inhibitor) was deposited on a filter paper, accumulating the haemolymph from 100 flies on the same paper. For the experiment to detect the presence of bacteria in the haemolymph, contamination between flies was avoided by washing the needle between each extraction first with one aliquot of protease inhibitor, followed by ethanol and a second, clean, aliquot of inhibitor, before refilling with a third aliquot of inhibitor.

### [^14^C]-TCT quantification

Haemolymph extracts or 1µL drops of a dilution series of the [^14^C]-TCT solution were deposited on Whatman filter paper, which was then exposed to a Phosphor Imager Screen (GE Healthcare) for 2 weeks at room temperature. Images were generated with a Typhoon Trio Phosphor Imager (GE Healthcare) and quantified with ImageQuant TL.

### Elicitor injection

PGN, TCT and water were injected using a Nanodrop microinjector (Nanoject™) [44]. Flies were pricked in the thorax with a glass needle containing the elicitor solution and 13nL was injected.

### Accession numbers

The Flybase (http://www.flybase.org) accession numbers for genes mentioned in this study are:

*Attacin A* (CG10146), *Cecropin A* (CG1365), *Defensin* (CG1385), *Diptericin* (CG12763), *Drosocin* (CG10816), *Drosomycin* (CG10816), *Gal* (CG9092), *Metchnikowin* (CG8175), *PGRP-LB* (CG14704), *PGRP-LC* (CG4432), *Pirk* (CG15678) and *Relish* (CG11992).

## Supporting Information

Figure S1 AMPs belonging to all families are specifically induced by Gram-negative bacteria upon genital infection. Expression profile of *Attacin A (AttA), Drosocin (Drc), Metchnikowin (Mtk), CecropinA (CecA), Defensin (Def)* and *Drosomycin (Drs)* in flies collected 18h after GI with various bacteria: *M.lut.: M. luteus, B.sub.: B. subtilis, Ecc15: E. carotovora*. Data are the mean of 4 repeats, error bars are standard errors.
(0.20 MB TIF)

Figure S2 Immune response to genital infections is also observed in unmated males. RT-qPCR analysis of *Dpt* expression in unmated or mated wild-type male flies collected 6h after GI infection with *Ecc15*. Males were collected a few hours after eclosion and left for 3 days either in the absence or presence of females, prior to infection. *Dpt/rpL32* ratios are shown normalised to expression 6h after GI in mated flies. Data are the mean of 3 repeats and error bars show standard error. UC - Unchallenged.
(0.04 MB TIF)

Figure S3 Local expression of *Defensin* and *Diptericin*, upon genital infection, is controlled by the Imd pathway. RT-qPCR analysis of *Dpt* and *Def* expression in the genital tract of wild-type (Or, white bars) and *Relish^E20^* (*Rel*, black bars) flies, 6h after deposition of TCT on the genital plate. Data are the mean of 3 repeats and error bars show standard error. UC - Unchallenged.
(0.08 MB TIF)

Figure S4 Similar *Diptericin* expression is induced by genital deposition of TCT or injection of 1/1000th dilution of TCT. RT-qPCR analysis of *Dpt* expression in wild-type flies 6h after injection or deposition on the genital plate (genital) of TCT or PGN. For the injection, flies were challenged with 1/10^th^ to 1/10000^th^ serial dilutions of the solutions of TCT and PGN used for genital challenge (initial concentrations 5mmol.L^−1^ for PGN and 1mmol.L^−1^ for TCT). 1/10^th^–1/1000^th^ dilutions of PGN all elicited similar levels of *Dpt* expression upon injection, suggesting that they are saturating the immune response. *AMP/rpL32* ratios are shown normalised to expression 6h after genital challenge with TCT. Data are the mean of 3 repeats and error bars show standard error. UC - Unchallenged, ND - not determined.
(0.10 MB TIF)
