# Supplementary figures and images for: Long-Range Activation of Systemic Immunity through Peptidoglycan Diffusion in Drosophila
Source: PLoS Pathog. 2009 Dec 18;5(12):e1000694. doi: 10.1371/journal.ppat.1000694 (PMC2787014; doi:10.1371/journal.ppat.1000694)

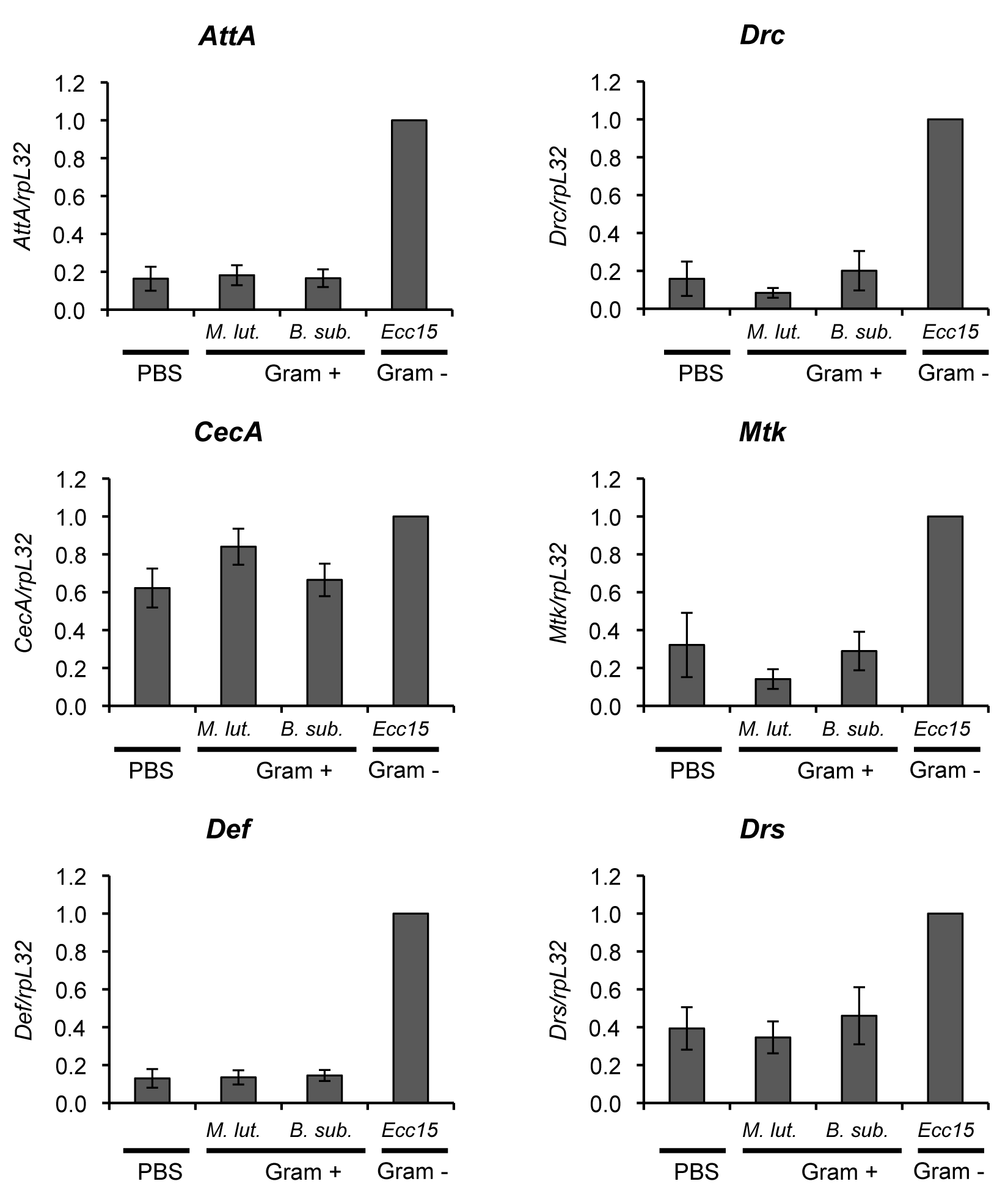

Supplement: Figure S1 — AMPs belonging to all families are specifically induced by Gram-negative bacteria upon genital infection. Expression profile of Attacin A (AttA), Drosocin (Drc), Metchnikowin (Mtk), CecropinA (CecA), Defensin (Def) and Drosomycin (Drs) in flies collected 18h after GI with various bacteria: M.lut.: M. luteus, B.sub.: B. subtilis, Ecc15: E. carotovora. Data are the mean of 4 repeats, error bars are standard errors. (0.20 MB TIF) [file ppat.1000694.s001.tif]

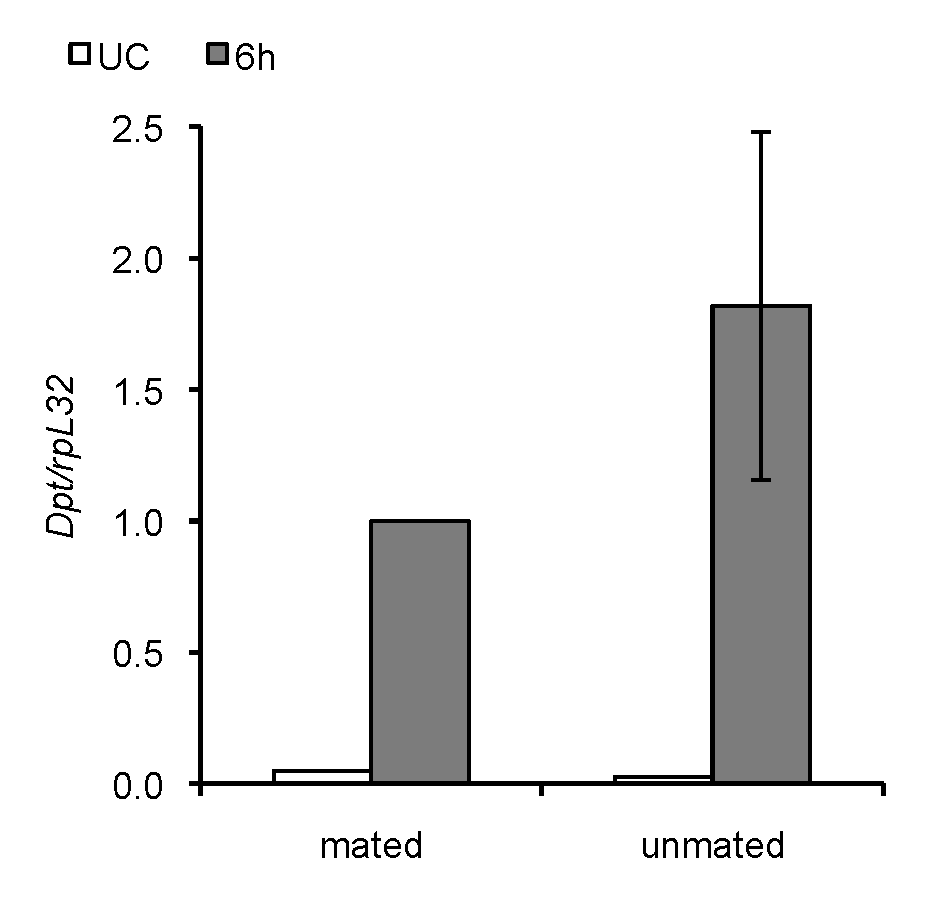

Supplement: Figure S2 — Immune response to genital infections is also observed in unmated males. RT-qPCR analysis of Dpt expression in unmated or mated wild-type male flies collected 6h after GI infection with Ecc15. Males were collected a few hours after eclosion and left for 3 days either in the absence or presence of females, prior to infection. Dpt/rpL32 ratios are shown normalised to expression 6h after GI in mated flies. Data are the mean of 3 repeats and error bars show standard error. UC - Unchallenged. (0.04 MB TIF) [file ppat.1000694.s002.tif]

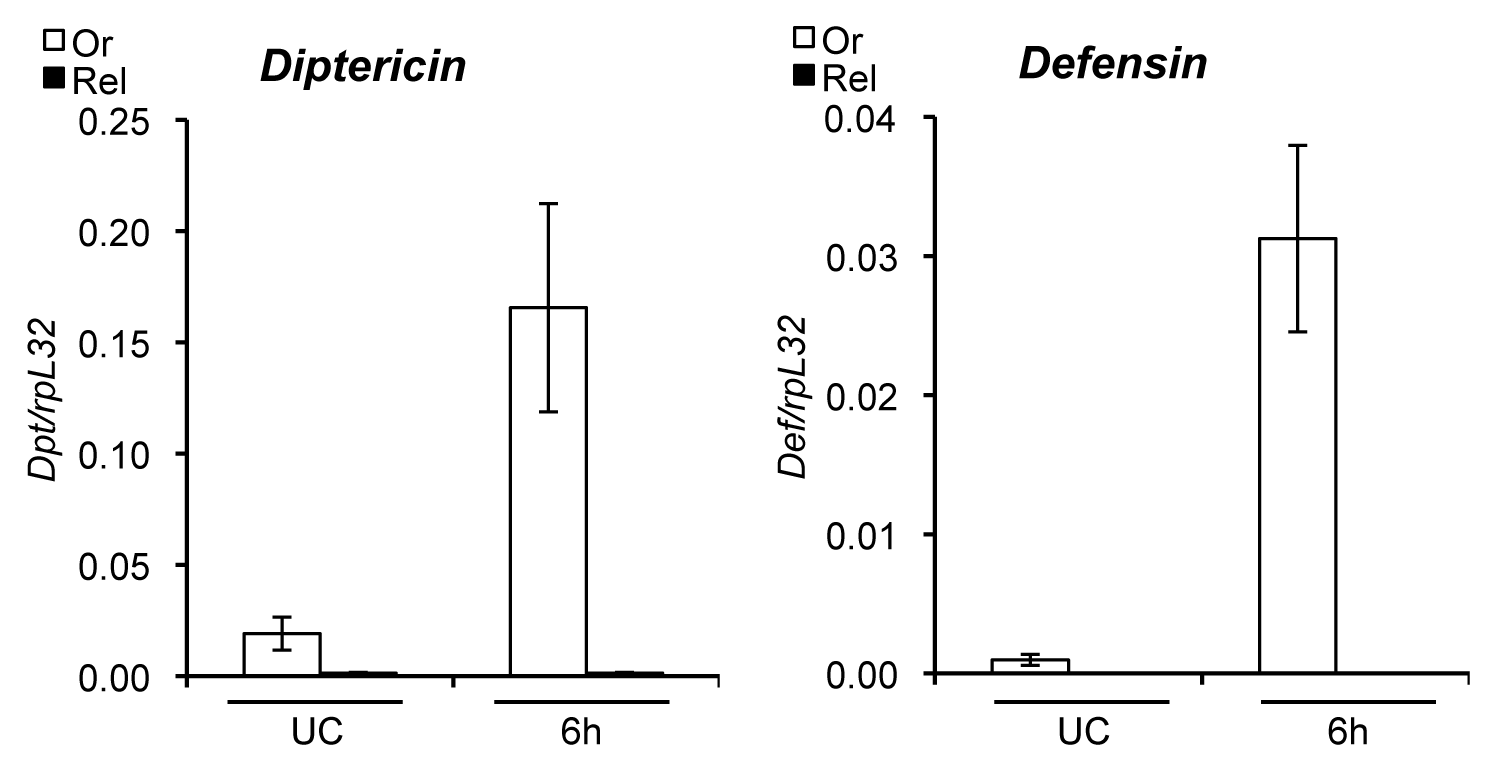

Supplement: Figure S3 — Local expression of Defensin and Diptericin, upon genital infection, is controlled by the Imd pathway. RT-qPCR analysis of Dpt and Def expression in the genital tract of wild-type (Or, white bars) and RelishE20 (Rel, black bars) flies, 6h after deposition of TCT on the genital plate. Data are the mean of 3 repeats and error bars show standard error. UC - Unchallenged. (0.08 MB TIF) [file ppat.1000694.s003.tif]

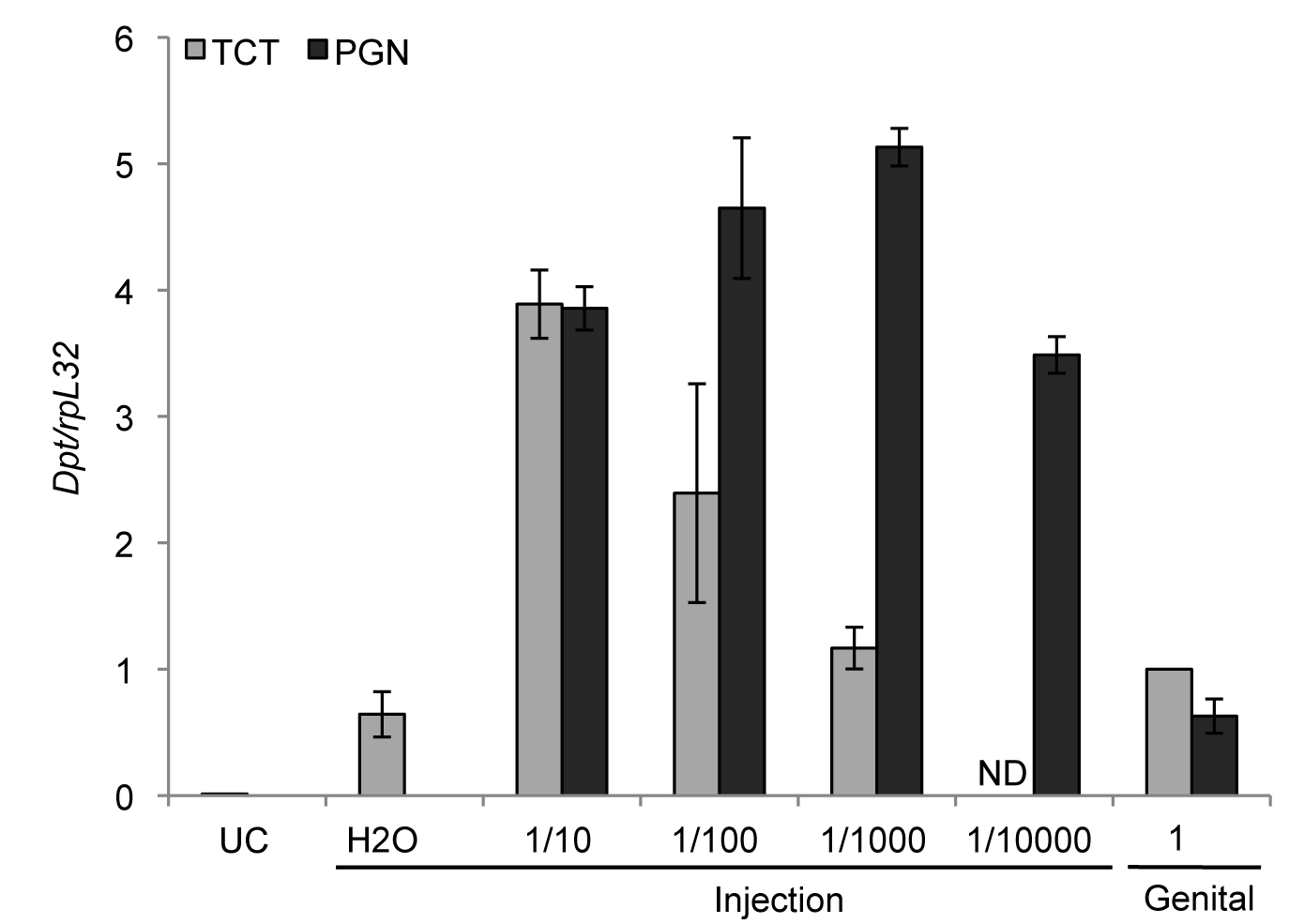

Supplement: Figure S4 — Similar Diptericin expression is induced by genital deposition of TCT or injection of 1/1000th dilution of TCT. RT-qPCR analysis of Dpt expression in wild-type flies 6h after injection or deposition on the genital plate (genital) of TCT or PGN. For the injection, flies were challenged with 1/10th to 1/10000th serial dilutions of the solutions of TCT and PGN used for genital challenge (initial concentrations 5mmol.L−1 for PGN and 1mmol.L−1 for TCT). 1/10th–1/1000th dilutions of PGN all elicited similar levels of Dpt expression upon injection, suggesting that they are saturating the immune response. AMP/rpL32 ratios are shown normalised to expression 6h after genital challenge with TCT. Data are the mean of 3 repeats and error bars show standard error. UC - Unchallenged, ND - not determined. (0.10 MB TIF) [file ppat.1000694.s004.tif]
